# Risk factors for hospital readmission in older adults within 30 days of discharge – a comparative retrospective study

**DOI:** 10.1186/s12877-020-01867-3

**Published:** 2020-11-11

**Authors:** Maria Glans, Annika Kragh Ekstam, Ulf Jakobsson, Åsa Bondesson, Patrik Midlöv

**Affiliations:** 1 Department of Clinical Sciences in Malmö, Center for Primary Health Care Research, Lund University, Clinical Research Center, Box 50332, 20213 Malmö, Sweden; 2 Department of Medications, Region Skåne Office for Hospitals in Northeastern Skåne, SE-291 85 Kristianstad, Sweden; 3 Department of Orthopaedics, Region Skåne Office for Hospitals in Northeastern Skåne, SE-291 85 Kristianstad, Sweden; 4 Department of Medicines Management and Informatics in Skåne County, SE-291 85 Kristianstad, Sweden

**Keywords:** Polypharmacy, Potentially inappropriate medication list, Patient readmission, Patient transfer, Aged, Aged 80 and over

## Abstract

**Background:**

The area of hospital readmission in older adults within 30 days of discharge is extensively researched but few studies look at the whole process. In this study we investigated risk factors related, not only to patient characteristics prior to and events during initial hospitalisation, but also to the processes of discharge, transition of care and follow-up. We aimed to identify patients at most risk of being readmitted as well as processes in greatest need of improvement, the goal being to find tools to help reduce early readmissions in this population.

**Methods:**

This comparative retrospective study included 720 patients in total. Medical records were reviewed and variables concerning patient characteristics prior to and events during initial hospital stay, as well as those related to the processes of discharge, transition of care and follow-up, were collected in a standardised manner. Either a Student’s *t*-test, χ^2^-test or Fishers’ exact test was used for comparisons between groups. A multiple logistic regression analysis was conducted to identify variables associated with readmission.

**Results:**

The final model showed increased odds of readmission in patients with a higher Charlson Co-morbidity Index (OR 1.12, *p*-value 0.002), excessive polypharmacy (OR 1.66, *p*-value 0.007) and living in the community with home care (OR 1.61, *p*-value 0.025). The odds of being readmitted within 30 days increased if the length of stay was 5 days or longer (OR 1.72, *p*-value 0.005) as well as if being discharged on a Friday (OR 1.88, *p*-value 0.003) or from a surgical unit (OR 2.09, *p*-value 0.001).

**Conclusion:**

Patients of poor health, using 10 medications or more regularly and living in the community with home care, are at greater risk of being readmitted to hospital within 30 days of discharge. Readmissions occur more often after being discharged on a Friday or from a surgical unit. Our findings indicate patients at most risk of being readmitted as well as discharging routines in most need of improvement thus laying the ground for further studies as well as targeted actions to take in order to reduce hospital readmissions within 30 days in this population.

## Background

Readmissions to hospital within 30 days of discharge pose a major risk to society. It is a risk not only for the well-being of patients, who risk exposure to infections, rise of adverse events, episodes of confusion as well as accidental injury through falls [1], but also for health economy issues [2, 3]. In Sweden, as in several other western countries, the frequency of 30-day readmission is approximately one in five [2–5]. Patients aged 65 or older account for approximately 56% of these early readmissions and close to 60% of the associated costs [3]. Since previous studies have shown that 27% of readmissions within 30 days are preventable [6], attempts at decreasing the frequency, especially in older adults, are called for. In Sweden, the national goal is to decrease, by 10%, in patients 65 years and older, the frequency of readmission to hospital within 30 days of discharge. To achieve this goal a better understanding of the underlying causes of early readmissions is required.

### Risk factors of readmission to hospital within 30 days

According to previous studies, the frequency of readmission to hospital within 30 days of discharge is higher for patients with several comorbidities [5, 7] as well as for those with certain medical conditions such as congestive heart failure, pneumonia and chronic obstructive pulmonary disease [2, 3]. A rise in frequency has been further observed with the number of previous discharges [2, 5, 7], male sex [2, 5], the number of medications used [8, 9], age [2, 3, 5] and length of stay at initial hospital visit [2, 5, 7].

### Medication without harm

In 2017 the World Health Organization (WHO) initiated its third global patient safety challenge; Medication Without Harm. The goal is to globally reduce the level of severe, avoidable harm related to medication by 50% over 5 years [10]. The primarily targeted areas are high-risk situations, including high-risk medications, polypharmacy and transitions of care [10]. These areas have, individually, also been indicated to play a role concerning readmission to hospital within 30 days, especially in older adults [5, 9, 11].

According to Forster et al. [11], 11% of patients experience adverse drug events (ADEs) within 30 days of discharge from hospital, of which approximately one third can be prevented. The risk of experiencing ADEs increase with age as a result of the pharmacokinetic and pharmacodynamic changes that occur [12–15].

Another risk factor for ADEs is polypharmacy, which is also more common with increasing age due to a greater number of comorbidities [12]. Polypharmacy is commonly defined as the daily intake of five or more prescription drugs [16] and, according to the Swedish National Board of Health and Welfare (SNBHW), this is also the average medication use among older Swedish adults (75 years and older) [17]. Approximately 12% in this population group use 10 or more medications on a regular basis [17], often referred to as excessive polypharmacy [16], which even further increases their risk of ADEs.

Previous research has shown that medication reconciliation and medication review during a hospital visit can reduce the number of ADEs [18, 19] and the use of potentially inappropriate medications (PIMs) in older patients [18, 20]. There are several lists of PIMs in use internationally, Beers criteria [21] perhaps being the most widely used. However, since several of the drugs listed in Beers criteria are unavailable in Europe, criteria corresponding to the European drug formularies, such as the “Swedish indicators of good medication therapy in the elderly” [22], have been developed. The Swedish indicators include support to prescribers in medication choices within specific diagnoses as well as drug specific indicators such as explicit lists of PIMs and fall-risk increasing drugs (FRIDs). The indicators also include a recommendation to avoid polypharmacy, especially the use of three or more psychoactive medications (including antidepressants, sedatives, hypnotics and antipsychotic medications).

Providing patients, their general practitioner (GP) and, if needed, municipal care nurse, with a discharge summary, including a medication report and an updated medications list, has been shown to decrease the number of medication errors and ADEs experienced after discharge [23–26]. This makes it a vital part of the transition of patients from hospital to primary care [10]. Since ADEs have been shown to lead to hospital admission [12–14, 27–29], as well as hospital readmission [30–32], there is cause to hypothesise that improving medication reconciliation and medication review, as well as information transfer regarding medication at discharge, could be pertinent to reducing readmission to hospital, especially in older patients using multiple medications.

Several researchers have identified factors related to processes of transition from hospital to primary care as being important in early hospital readmission [25, 33–35]. This suggests that improvements can be made nationally and actions be taken locally to reduce the proportion of patients readmitted, all in accordance with the WHO Medication Without Harm challenge [10].

The area of readmission of older adults to hospital within 30 days of discharge is, as shown, extensively researched and previous results point out risks within several aspects of the area. There are, however, few studies looking at the whole process.

In this study we investigated risk factors related, not only to patient characteristics prior to and events during initial hospital stay, but also to the processes of discharge, transition of care and follow-up. By doing so we aimed to identify patients at most risk of being readmitted as well as processes in greatest need of improvement, the goal being to find ways to help reduce early readmissions in this population.

## Methods

### Setting

This study was conducted at the hospital in Kristianstad (255 beds), one of ten hospitals in the county of Skåne in the south of Sweden. The hospital receives emergency patients as well as elective and out-patient appointments from the municipalities surrounding Kristianstad. In 2017 the rate of 30-day hospital readmission in patients 65 years and older was 18% [36].

In Sweden the provision of healthcare, primary and hospital, is the responsibility of the regions. Nursing care for the elderly, on the other hand, in their homes or in nursing homes, is provided by the local municipality in collaboration with physicians from the region, primarily GPs. If patients are in need of intensified municipal care after discharge from hospital these activities are planned together with the municipality before discharge. This can result in the initiation of, for example, help with daily activities, cooking, shopping or cleaning (home care), help with dispensing medications or dressing wounds in the patient’s home (home healthcare) or the decision to move the patient into a nursing home.

At discharge from hospital the patient and, if applicable, nurse in municipal care, are provided with a discharge summary containing brief information about the cause of hospitalisation, what happened during the hospital stay and any follow-up plans after discharge. Also included in the discharge summary is a medications list as well as a medication report summarising which medication changes were made during the hospital stay and why. The document is sent, by post, to the primary care centre where it is scanned into the electronic medical records. Information transfer to the primary care centre also occurs via a medical case history. This is a more detailed document on the hospital stay not including a medication report or medications list. If follow-up is needed by the GP, a referral is to be sent to the primary care centre.

The medications list in the discharge information is derived from the full list of medications prescribed within the electronic hospital medical record. Since this list is not always up to date medication reconciliation needs to be performed and documented at admission to hospital. During the hospital stay it is possible to activate only current prescriptions but at discharge it is pertinent to assure that the medications list in the discharge information is consistent with the one used during the hospital visit.

When patients, aged 65 years and older, are admitted to a hospital in Skåne county the attending doctor should perform medication review as well as medication reconciliation [37]. Clinical pharmacists can be of service in performing these tasks but they are primarily focused on patients aged 75 years and older using five medications or more [38].

### Design and study population

This comparative retrospective study included 720 adults, aged 65 years and older, who were admitted to the hospital in Kristianstad in 2017. The study group (*n* = 360) were subsequently readmitted to either of the hospitals in the county within 30 days of discharge whereas the comparison group (*n* = 360) was not. First admissions were evenly distributed over the year with 30 patients per group and month being randomly selected and henceforth included. The last groups to be included were initially admitted in December 2017 and follow-up ended as the last of the participants was readmitted, within 30 days of initial discharge. Electronic hospital medical records and paper printouts from the electronic medical records in primary care were reviewed. Patients who had a planned readmission, patients deceased during initial hospital stay and patients who went home against medical advice were excluded from the study as were patients who were readmitted the same day as being discharged. Only the first of multiple unplanned 30-day readmissions was considered. Patients could only occur once in the study, either in the study group or in the comparison group.

To calculate sample size it was assumed that the amount of patients treated with potentially inappropriate medication regimens in the study group was 40% as compared to 30% in the comparison group, the amounts being based on previous studies [13, 27, 28]. This amounted to a desired sample size of at least 356 patients per group when aiming at a power of 80% and *p* < 0.05.

Outcomes of primary interest were the proportion of patients with polypharmacy and excessive polypharmacy as well as the proportion of patients using PIMs, FRIDs or three or more psychoactive drugs as specified by the SNBHW [22] upon discharge of the initial hospital stay. The consistency of information transfer at discharge was another factor of primary interest.

### Data collection

**Fig. 1:**
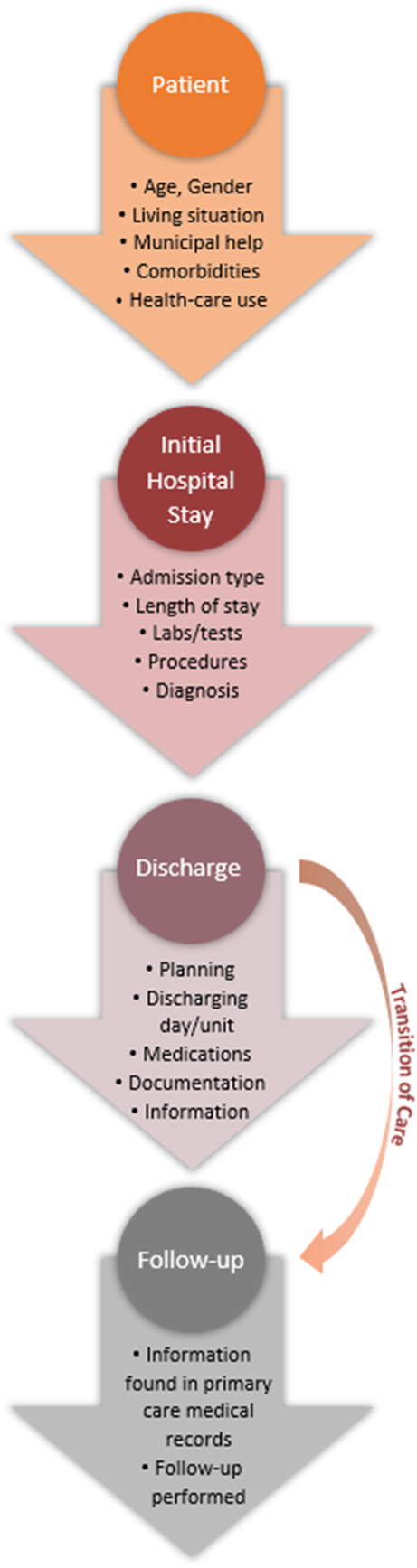
The chain of events covered in the data collection includes patient characteristics prior to and events during the initial hospital stay as well as factors concerning the processes of discharge, transition of care and follow-up

In an attempt to cover the whole chain of events, as depicted in Fig. 1, an array of variables concerning patient characteristics prior to and events during the initial hospital stay as well as factors concerning the processes of discharge, transition of care and follow-up were collected in a standardised manner in the medical records by the first author (MG).

Comorbidities took into account active and previous medical problems stated in the hospital medical record at the time of initial discharge and were assessed using the age adjusted Charlson Comorbidity Index (CCI) [39, 40].

The documentation of a performed medication reconciliation, by a doctor or a pharmacist, was compared between groups as was the consistency between the list of medications used during the hospital stay and the one included in the discharge information.

All medications prescribed at discharge were documented and the number of medications used was calculated in a standardised manner. If the same medication was given daily, as well as per request or if the same medication was given in different dosage forms, they counted as two different medications. If medications with the same active ingredient were prescribed for daily intake in different doses it counted as one medication. Obvious duplicates counted as one medication.

Polypharmacy was defined as the daily use of five or more medications and excessive polypharmacy as the daily use of ten or more medications, according to the most common definitions as shown by Masnoon et al. [16].

Medical changes made during the hospital stay were determined by assessing the medications list used as well as documentation made in the medical record. The changes made and information found were subsequently compared with the changes documented in the discharge information and medical case history as well as with the information put forward in the medication report.

Medications were further analysed to determine use of PIMs, FRIDs or three or more psychoactive drugs as specified by the SNBHW [22] as well as medications commonly known to cause ADEs.

### Data analysis

Descriptive statistics for each variable were calculated using IBM SPSS Statistics ver. 26. Data for continuous variables are presented as mean and standard deviation (SD), and categorical data as proportions (%). Continuous data was compared between groups with an unpaired Student’s *t*-test while a χ^2^-test or Fishers’ exact test was used for comparison of categorical data. A multiple logistic regression analysis (manual backward) was conducted to identify variables associated with readmission. Readmission (0 = No; 1 = Yes) was used as the dependent variable. The following variables were used as independent variables in the multiple regression analysis: Charlson Comorbidity Index (age adjusted); Number of hospitalisations 12 months; Living in own home, alone; Living in own home, with spouse/other; Living in own home with home care; Help with medications from nurse; Multi-dose drug dispensing; Using walking aid; Chronic ischaemic heart disease; Leg ulcer; Atrial fibrillation; History of embolic disease; Blood haemoglobin level at discharge; Length of stay 5 days or longer; Major surgery; Polypharmacy; Excessive polypharmacy; Number of FRIDs; Use of Diuretics; Use of anticoagulants; Dosages adjusted; Medications withdrawn; Day of discharge and Discharging unit. The independent variables “Day of discharge” and “Discharging unit” (ordinal data), were dummy coded with “Monday-Thursday” and “Internal medicine” respectively as reference. In each step of the manual backward logistic regression the variable with the highest *p*-value was withdrawn from the model until all remaining variables had a *p*-value of < 0.05. The regression model was controlled for the variables gender and type of admission (age adjusted for in the Charlson comorbidity index). A goodness-of- fit test was carried out on the regression model using the Hosmer and Lemeshow goodness-of-fit test [41] and Nagelkerke R2. Significance was specified as *p* < 0.05.

## Results

### Patient characteristics prior to admission

A total of 720 patient hospital records were reviewed with 360 patients in each group. There were no significant differences between groups in regard to age or gender as shown in Table 1. However, the proportion of patients aged 75 years or older was significantly higher in the study group (72%) as compared to the comparison group (62%, *p*-value 0.003). Patients readmitted to hospital within 30 days of discharge were in poorer health overall as shown by a higher healthcare utilisation, a higher comorbidity score and a greater dependency on municipality help prior to admission (Table 1). Several comorbidities were shown to be significantly more common in the study group (Table 2).

**Table 1:** Patient characteristics prior to admission

Characteristic	Comparison group (***n*** = 360)	Readmitted < 30 days (***n*** = 360)	***p***-value
Age^a^, Mean (SD)	78 (8)	80 (8)	0.072^b^
Female, %	52	49	0.333^c^
Number of hospitalisations 12 months, Mean (SD)	1.4 (1.0)	2.0 (1.3)	**< 0.001**^b^
Number of visits to ER 6 months, Mean (SD)	0.3 (0.7)	0.5 (0.9)	**0.001**^b^
Charlson Comorbidity Index, Mean (SD)	6 (3)	8 (3)	**< 0.001**^b^
***Living situation and disability***			
Nursing home, %	11	13	0.299^c^
Own home, with spouse/other, %	53	43	**0.005**^c^
Own home, alone, %	36	44	**0.028**^c^
Own home with home care, %	16	33	**< 0.001**^c, d^
Help with activities in daily life, %	12	19	**0.007**^c^
Own home with home healthcare, %	10	19	**0.002**^c, f^
Help with medication from nurse, %	16	25	**0.004**^c^
Multi-dose drug dispensing, %	14	22	**0.005**^c, d^
Using walking aid, %	44	60	**< 0.001**^c, e^
Using wheelchair, %	8.4	16	**0.003**^c, d^

*Abbreviations*: *ER* Emergency Room, *SD* Standard Deviation

^a^Variables included in the Charlson Comorbidity Index ^b^Students t-test ^c^χ^2^-test ^d^Internal missing 0.3–0.4% ^e^Internal missing 3.1% ^f^Internal missing 8.9%

Significant *p*-values are indicated in bold.

**Table 2:** Active and previous comorbidities as stated in the hospital medical records

Diagnosis	Comparison group (***n*** = 360)	Readmitted < 30 days (***n*** = 360)	***p-***value
Chronic ischaemic heart disease, %	24	34	**0.007**^b, d^
Myocardial infarction^a^_,_ %	22	24	0.535^b, d^
Congestive heart failure^a^, %	26	33	**0.033**^b^
Peripheral vascular disease^a^, %	7.8	9.2	0.503^b^
CVA^a^ or TIA^a^, %	29	27	0.507^b^
Dementia^a^, %	13	17	0.141^b, d^
Chronic obstructive pulmonary disease^a^, %	13	26	**< 0.001**^b^
Connective tissue disease^a^_,_ %	22	20	0.463^b^
Peptic ulcer disease^a^, %	16	23	**0.038**^b^
Mild liver disease^a^, %	0.6	2.2	0.063^c, e^
Severe liver disease^a^, %	0.0	1.1	0.124^c^
Diabetes mellitus^a^, %	17	21	0.130^b^
Hemiplegia^a^, %	2.5	1.9	0.613^c^
Kidney failure stadium IV^a^, %	4.5	14	**< 0.001**^b, e^
History of cancer, %	31	44	**< 0.001**^b^
Solid tumour^a^, %	14	26	**< 0.001**^b^
Metastatic tumour^a^, %	3.9	14	**< 0.001**^b^
Leukaemia^a^, %	1.9	1.9	1.000^c^
Lymphoma^a^, %	0.8	3.3	**0.019**^c^
AIDS^a^, %	–	–	–
Leg ulcer, %	3.3	7.5	**0.014**^b^
Atrial fibrillation, %	28	37	**0.011**^b^
History of embolic disease, %	8.3	14	**0.013**^b^

*Abbreviations*: *CVA* Cerebrovascular accident, *TIA* Transient ischaemic attack, *SD* Standard Deviation

^a^Variables included in the Charlson Comorbidity Index ^b^χ^2^-test ^c^Fishers’ exact test ^d^Internal missing 0.1–0.3% ^e^Internal missing 0.7–1.1%

Significant *p*-values are indicated in bold.

### Initial hospitalisation

While readmitted patients did not have a significantly longer mean initial hospital stay, the proportion of patients who stayed for 5 days or longer was higher in the study group (Table 3). Patients subsequently readmitted were in greater need of care planning before discharge (29% in the study group as compared to 21% in the comparison group, *p*-value 0.017) and discharge on a Friday was shown to increase the risk of being readmitted as was being discharged from a surgical unit (Table 3). The latter risk increased further if the initial hospitalisation was emergent as compared to elective (25% as compared to 17%, *p*-value 0.010). On the other hand, patients who had undergone major surgery at initial hospitalisation were shown to have a significantly lower risk of being readmitted within 30 days of discharge (Table 3). An equal result was attained when excluding elective initial hospitalisations. Out of the 650 patients with an emergent initial hospitalisation, a total of 56 patients (17%) had undergone major surgery in the study group as compared to 74 patients (23%) in the comparison group (*p*-value 0.040).

**Table 3:** Variables related to the initial hospitalisation and discharge

Variable	Comparison group (***n*** = 360)	Readmitted < 30 days (***n*** = 360)	***p***-value
Emergent hospitalisation, %	88	93	0.058^c^
Hospitalisation related to cancer diagnosis, %	6.9	11	0.051^c^
Length of stay, Mean (SD)	6.9 (7.2)	7.8 (5.2)	0.063^b^
Length of stay 5 days or longer, %	53	66	**< 0.001**^c^
***Laboratory and test results (last result before discharge)***			
Plasma sodium level, mmol/L, Mean (SD)	140 (3.7)	139 (3.8)	0.267^b, e^
Plasma potassium level, mmol/L, Mean (SD)	4.0 (0.4)	4.0 (0.4)	0.815^b, e^
Blood haemoglobin level, g/L, Mean (SD)	124 (19.2)	119 (18.1)	**< 0.001**^b, e^
Relative eGFR^a^, Mean (SD)	59 (18.2)	55 (20.9)	**0.006**^b, d^
Body Mass Index < 22, kg/m^2^, %	30	35	0.153^c, f^
***Procedure performed during hospital stay***			
Minor surgery/diagnostic procedure, %	33	37	0.241^c^
Major surgery, %	31	21	**0.002**^c^
***Variables concerning discharge***			
Discharged Monday-Thursday, %	70	66	0.230^c, d^
Discharged Friday or day before holiday, %	19	27	**0.012**^c, d^
Discharged weekend or holiday, %	11	6.7	0.064^c, d^
Discharged from Internal medicine, %	56	54	0.600^c^
Discharged from General surgery, %	21	28	**0.046**^c^
Discharged from Infection, %	7.2	6.1	0.550^c^
Discharged from Orthopaedics, %	11	8.9	0.320^c^
Discharged from Gynaecology/ENT unit, %	4.2	3.3	0.556^c^

*Abbreviations*: *ENT* Ear Nose Throat, *eGFR* Estimated Glomerular Filtration Rate, *SD* Standard Deviation

^a^Variables included in the Charlson Comorbidity Index ^b^Student’s t-test ^c^χ^2^-test ^d^Internal missing 0.4–1.7% ^e^Internal missing 3.2–4.6% ^f^Internal missing 13.5%

Significant *p*-values are indicated in bold.

As shown in Table 4, readmitted patients used a significantly larger amount of medications overall, including FRIDs and some medications known to cause ADEs. In readmitted patients, the most common regularly used medications at discharge were paracetamol (40%), furosemide (35%) and metoprolol (30%) whereas the comparison group used low dose acetylsalicylic acid (33%), metoprolol (32%) and paracetamol (32%).

**Table 4:** Medications, medication review and transfer of medication information at discharge

Variable	Comparison group (***n*** = 360)	Readmitted < 30 days (***n*** = 360)	***p***-value
Number of medications in total, Mean (SD)	9.0 (4.9)	11 (5.3)	**< 0.001**^d^
Number of daily medications, Mean (SD)	7.2 (3.8)	8.8 (4.1)	**< 0.001**^d^
Number of medications as needed, Mean (SD)	1.8 (1.8)	2.4 (2.1)	**< 0.001**^d^
Polypharmacy^a^, %	74	86	**< 0.001**^e^
Excessive polypharmacy^b^, %	24	42	**< 0.001**^e^
***Medications often connected to ADEs***			
Diuretics, %	41	52	**0.002**^e^
Antihypertensive medications (not diuretics), %	68	72	0.256^e^
Anticoagulants (including LMWH), %	27	34	**0.023**^e^
Anti-platelet drugs, %	39	32	0.052^e^
Insulin, %	9.7	14	0.066^e^
Digoxin, %	4.2	4.2	1.000^e^
Opiates, %	31	37	0.098^e^
***Potentially inappropriate medication regimens***^***c***^			
No of PIMs, Mean (SD)	0.17 (0.41)	0.16 (0.44)	0.662^d^
No of FRIDs, Mean (SD)	3.24 (2.24)	3.64 (2.13)	**0.014**^d^
Use of three or more psychoactive drugs, %	10.0	9.2	0.704^d^
***Medication changes made during hospital stay***			
Changes in medications made during stay, %	84	88	0.110^e^
New medications started, %	79	75	0.249^e^
Medications withdrawn, %	23	31	**0.024**^e^
Dosages adjusted, %	24	34	**0.003**^e^
Medication changes in discharge summary, %	58	51	0.061^e, f^
Medication changes in medical case history, %	52	47	0.180^e^
Medication report correct, %	56	50	0.124^e^

*Abbreviations*: *LMWH* Low Molecular Weight Heparin, *PIM* Potentially Inappropriate Medication, *FRID* Fall Risk Increasing Drug, *SD* Standard Deviation

^a^Defined as the daily use of five medications or more ^b^Defined as the daily use of 10 medications or more ^c^According to the Swedish National Board of Health and Welfare ^d^Students t-test^e^χ^2^-test ^f^Internal missing 0.1%

Significant *p*-values are indicated in bold.

Medication reconciliation was only documented to a small extent overall (38.1% in the study group as compared to 38.3% in the comparison group, *p*-value: 0.939). The proportion of patients receiving a medication review performed by a clinical pharmacist was also small but significantly larger in the study group than in the comparison group (23% as compared to 15%, *p*-value: 0.008). However, after solely analysing patients aged 75 years and older, who are the ones mainly targeted by clinical pharmacists at the hospital, there was no significance between groups (32% in the study group as compared to 24% in the comparison group, *p*-value: 0.058).

### Discharge process, transition of care and follow-up

Patients subsequently readmitted to hospital within 30 days of discharge more often had medications withdrawn or dosages adjusted during their initial hospital stay (Table 4). These medical changes were generally inadequately documented in the discharge summary but even though data points towards slightly poorer documentation in the study group, the difference was not significant.

Due to restrictions in access to medical journals from privately owned primary care centres, only about 50% of primary care medical records were available for analysis. Data points towards no significant difference between groups in the transfer of discharge summaries to primary care centres (68% transferred in the study group as compared to 71% in the comparison group, *p*-value: 0.397). However, when performing the same analysis solely in patients in need of follow-up by the GP, according to the medical case history, the discharge summary was less frequently found in the electronic journal at the primary care centre in patients subsequently readmitted.

Only 79% of discharge summaries were found in primary care electronic medical records in readmitted patients whereas 93% were found in patients that were not readmitted (*p*-value 0.015). There was a significantly lower rate of follow-up performed by the GP in the study group (32%) as compared to in the comparison group (44%, *p*-value: 0.013). This difference persisted in patients in documented need of follow-up. In this cohort, 40% of readmitted patients had a follow-up by their GP and 60% of patients that were not readmitted (*p*-value: 0.018).

### Multiple logistic regression

The multiple logistic regression analysis identified several significant risk factors concerning readmission to hospital within 30 days of discharge in this population. The final model (Table 5) shows increased odds of readmission in patients with several comorbidities using 10 or more daily drugs and living in the community with home care. The odds of being readmitted within 30 days increase if length of initial hospital stay is 5 days or longer and there is a significant increase in odds if discharged on a Friday or from a surgical unit. There are, however, decreased odds of being readmitted after major surgery but this preventive effect disappears when the age-adjusted Charlson Comorbidity Index reaches ≥5 (OR 0.63, CI95% 0.37–1.07, *p*-value: 0.085). No significance was shown between groups regarding use of neither PIMs, FRIDs or three or more psychoactive drugs nor medications commonly associated with ADEs.

**Table 5:** Variables associated with readmission to hospital within 30 days of discharge^a^

Variable	OR	95% CI	***p***-value
Gender	0.89	0.63–1.25	0.500
Emergent admission	1.43	0.73–2.77	0.297
Charlson Comorbidity Index	1.12	1.04–1.20	**0.002**
Number of hospitalisations 12 months	1.41	1.19–1.68	**< 0.001**
Length of stay 5 days or longer	1.72	1.18–2.49	**0.005**
Day of discharge (reference: Monday-Thursday)			**0.007**
Friday/Day before weekend/holiday	1.88	1.24–2.87	**0.003**
Weekend/Holiday	0.83	0.44–1.54	0.551
Discharging unit (reference: Internal medicine)			**0.004**
General surgery	2.09	1.34–3.24	**0.001**
Infection	0.54	0.26–1.13	0.101
Orthopaedics	1.02	0.53–1.96	0.949
Gynaecology/Ear Nose Throat	1.47	0.55–3.93	0.442
Living in own home with home care	1.61	1.06–2.45	**0.025**
Major surgery performed during Index	0.59	0.37–0.94	**0.027**
Excessive polypharmacy^b^	1.66	1.15–2.40	**0.007**

*Abbreviations*: *CI* Confidence Interval

^a^Adjusted for gender, type of admission and age (within the age-adjusted Charlson Comorbidity Index) ^b^Defined as the daily use of 10 medications or more

Hosmer Lemeshow goodness of fit test *p*-value: 0.457. Nagelkerke *R*^2^: 0.228.

Significant *p*-values are indicated in bold.

## Discussion

This comparative retrospective study identified older adults of poor health, living in the community dependent on home care and using 10 or more medications on a regular basis as having increased odds of being readmitted to hospital within 30 days of discharge, especially if discharged on a Friday or from a surgical unit.

Many researchers have studied this area before but, while most previous studies focus on one part of the process, we looked at the whole process in detail. We involved all aspects of the process including variables related, not only to patient characteristics prior to and events during initial hospital stay, but also to the processes of discharge, transition of care and follow-up. In order to further generalise our findings we included patients with all-cause admission, from all departments of the hospital, evenly distributed throughout the year.

We found that patients readmitted to hospital within 30 days of discharge had a higher comorbidity index, were more frequently hospitalised in the last 12 months and more often stayed at the hospital for 5 days or more at initial hospitalisation than did patients that were not readmitted. This has been previously shown in several studies [2, 5, 7] and suggests that these patients, poorer of health, are the ones to target in actions trying to reduce early readmission in older adults.

Polypharmacy is another previously known risk factor concerning readmission to hospital. In 2018 Basnet et al. [9] performed a large (25,190 patients) retrospective cohort study in patients aged 65 years and older showing a 1.04 times increase in odds of being readmitted with every added medication. Picker et al. [8] performed a similar study, albeit in a smaller (5507 patients) and younger population (> 18 years), describing a 1.26 times increase in odds of being readmitted if using six medications or more on a regular basis. Our results did not show increased odds of being readmitted to hospital due to polypharmacy, defined as the use of five or more medications on a daily basis. We did, however, find that excessive polypharmacy, defined as the use of ten or more daily medications, increased the odds of being readmitted to hospital within 30 days 1.66 times. This implies that excessive polypharmacy is a major risk factor regarding readmission to hospital within 30 days of discharge in older adults of poor health.

Adding to previous knowledge is our identification of older adults living at home with home care before admission as having increased odds of early hospital readmission, as compared to those living in the community not depending on help from the municipality or living in nursing homes. Previous research concurs with a variation in proportion of readmission when considering the care setting before and after hospitalisation [42, 43] but these studies are mainly focused on the discharge destination whereas we show the importance of considering the care setting before admission. Older adults living in the community with home care are often debilitated and, even though generally not as frail as those living in nursing homes, in need of supervision and help in daily activities. Whereas older adults living in nursing homes have close access to nurses and doctors, those living in their own home with home care are often dependent on relatives or other relations, something that need to be taken into consideration in planning transitions of care. Several studies have shown that actions focused on improving discharge planning, patient education and follow-up are found to be most effective in reducing readmissions [44–47] and our results give reason to hypothesise that such actions would benefit from focusing on older adults of poor health, using ten medications or more and living in the community with home care. Further studies are, of course, needed to draw such conclusions.

In contradiction to the above-mentioned studies, Field et al. [48] could not find a decrease in readmissions when patients aged 65 years and older were followed-up by their GP within a week of discharge. This concurs with our results where we could not show that lack of follow-up by the GP after discharge was associated with increased odds of readmission. However, due to limited access to medical records from privately owned primary care facilities, no real conclusions can be drawn from these results. Neither can we be certain that the non-significant difference between groups in regard to transfer of information to the primary care centre is correct and there is no way of telling if the patient actually received or took part of the contents of their discharge summary or not. Previous studies do, however, show that discharge summaries are often lacking in content and quality [49, 50] as is the transfer of these to the next caregiver [50]. Even though the discharge summary, medication report and medications list are considered to be helpful to the GPs, their use of the information provided is often lacking due to the uneven quality as well as the discrepancies found in the medications lists included [49–51]. These deficits lead to risks of medication errors and ADEs for older patients in transitions of care [49, 52] and could be part of the reason as to why older adults living in the community with home care have increased odds of readmission as compared to those living in nursing homes.

According to Caleres et al. [49] discharge summaries from surgical departments are more often found to include discrepancies than those from other departments. This could, following our reasoning that these discrepancies could cause ADEs, be part of the explanation as to why patients discharged from surgical units were more at risk of being readmitted in the present study. Another factor could perhaps be that surgical departments more often discharge their patients on Fridays, something that, in itself, was shown to be a risk factor of readmission in this study. In 2002 Van Walraven et al. [53] showed that discharge on a Friday as compared to on a Wednesday increased the risk of being readmitted within 30 days. Au et al. [54], on the other hand, could not show a difference in readmission between patients discharged on Mondays, Wednesdays or Fridays while they showed a decreased risk of readmission when discharged at the weekend. Other studies [55, 56] showed no difference in risk of being readmitted after weekend discharge and nor did we. Van Walraven attributed the increased risk of being readmitted after being discharged on a Friday to the fact that there are more discharges taking place on this particular day of the week [53]. This is true also in Sweden where many units decrease their bed count and personnel over the weekend which could, in theory, result in forced discharges on Fridays. This could result in stressed and insufficient discharging procedures leading to an increased risk of readmissions; something that would need to be investigated further in order to draw conclusions.

Even if being discharged from a surgical unit increased the odds of readmission in our population, undergoing major surgery during initial hospitalisation did in fact decrease those odds. This preventive effect, however, disappeared as the Charlson Comorbidity Index increased, which is in agreement with results by Halfon et al. [7]. This was explained by Halfon et al. as being natural since surgeries in patients with fewer comorbidities are usually acute and less prone to major complications [7]. This was not investigated further in the present study.

Medication reconciliation and medication review performed by a clinical pharmacist have been shown to significantly decrease the number of medication related readmissions to hospital [30, 31]. Hypothesising that this would also be the case in all-cause readmissions in our population, these variables were included in the analysis. Unfortunately, a very low proportion of the population, in both groups, were subject to these actions, leading to difficulties in drawing conclusions from the result. But even though the lack of difference between groups could be caused by this lack of data it could also amount to the fact that medication reconciliation and medication review are indeed only effective in medication related hospital readmissions, something that was not looked at in this study.

Our results showed no significant difference between readmitted patients and those that were not readmitted in regard to the use of PIMs, FRIDs or three or more psychoactive drugs, which is in agreement with previous research [9, 57]. Other studies [58, 59], however, do show an association between the use of PIMs and an increased risk of being readmitted to hospital. In these studies the follow-up time was longer than 30 days implying that the use of PIMs may be a risk factor of readmission to hospital in the long run but not within 30 days. Furthermore, PIMs are mostly indicated to be associated with medication related admissions [27] and thereby, perhaps, with medication related readmissions. This was not investigated further in this study.

### Strengths and weaknesses

In this retrospective medical records study we examined two groups, equal in size, in order to find risk factors associated with readmission to hospital within 30 days of discharge. Patients’ initial hospital stays were evenly distributed over the year with 30 patients included per group and month thus decreasing the risk of seasonal differences contributing to risk. The fact that no patient occurred more than once contributed to finding as many different risk factors as possible in the sample.

This is a comprehensive study of readmissions including a large amount of variables, covering patient characteristics prior to and events during initial hospitalisation as well as processes of discharge, information transfer and follow-up. Data collection from medical records was structured and standardised instruments were used as needed thus making the study robust. However, even though a large amount of variables equal many factors both in patient and process that could be found to contribute to readmissions, there is also an increased risk of collinearity. Efforts have been made to avoid such problems in the multiple logistic regression model but there is still a risk that they exist and consequently affect the final result.

One limitation is the high degree of internal missing values in some variables leading to less trustworthy results and the fact that the study is set in a single hospital, which may lead to the results not being generalisable to other settings.

### Summary and further studies needed

In this study we aimed at finding risk factors associated with readmission to hospital within 30 days of discharge in older adults. The results show that patients of poorer health, using ten or more medications and living in the community dependent on municipal care are at greater risk of being readmitted and that readmissions more often occur after being discharged on a Friday or from a surgical unit.

As our data points towards the discharging process and information transfer being important in decreasing the risk of readmission within 30 days, further studies of how these processes could be improved, preferably in the view of doctors working at the hospital, would be very valuable in order to find ways to reduce the rate of readmission in this population.

Further studies are also needed in order to find out if potentially inappropriate medication regimens or other specific risk factors are associated with medication related readmissions to hospital in this population as well as to see if medication reconciliation and medication review can help reduce this risk.

## Conclusions

In older adults, those of poorer health, using ten or more medications regularly and living in their own home dependent on municipal care are at greater risk of being readmitted to hospital within 30 days of discharge. The odds of readmission increase after being hospitalised for 5 days or longer and if being discharged on a Friday or from a surgical unit.

Our findings indicate discharging routines in need of improvement as well as patients at most risk of being readmitted, laying the ground for further studies as well as targeted actions to take in order to reduce the proportion of hospital readmission within 30 days in this population.

## Data Availability

The datasets generated and/or analysed during the current study are not publicly available since sharing of data was not included in the approval from the ethics committee but are available from the corresponding author on reasonable request.

## Abbreviations

WHO: World Health Organization
ADE: Adverse Drug Event
GP: General Practitioner
SNBHW: Swedish National Board of Health and Welfare
PIM: Potentially Inappropriate Medication
FRID: Fall Risk Increasing Drug
CCI: Charlson Comorbidity Index
